# Intelligence

**Published:** 1810-04

**Authors:** 


### INTELLIGENCE.

M. Modeste Paroletti, has published a very long Memoir in the *Journal de Physique*, for the month of March last, of which we can only give the outline. It goes to excite the attention of the learned, to discover the nature of morbific exhalations, by the employment of colouring matters, as chemical re-agents hitherto little known, in order to throw light upon certain diseases.

Having observed that the bright scarlet colour of the American seed (*abrus precatorius*) changed into an ebony black, in the space of an hour, when held between the hands of his child, and placed in the bed at the time he lay ill with a putrid fever, he instituted an enquiry into the cause of this phenomenon. He found that the whole of the inside of these seeds became blackened, and that their natural colour was in part restored by the nitric acid. He tried in vain to re-produce the same phenomenon with the same patient.

These red seeds, exposed to the exhalation of putrefying animal substances, acquired a shade of dark red. Carbonic acid gas gave them in a few days a deep tinge, like the colour of wine; plunged into a mixture of three parts of hydrogen gas and two parts of azotic gas, they became of a blackish brown colour; but they took a very intense black colour, very nearly resembling that produced by the morbific virus, when they were immersed in a mixture of three parts of hydrogen, one of azote, and one of carbonic acid. Carbureted, sulphureted, and phosphureted hydrogen gas, appeared to him, when tried separately, to produce no action upon the red colour of the American seeds.

In reasoning upon these facts, M. Paroletti says, " It is probable that hydrogen, azote, and carbon, are among the number of constituent constituent principles of morbific miasmata; it is natural to believe that the nitric acid, which acts in a different way to what these three substances do, exerts a decomposing power upon them. This opinion is confirmed by theory, and by the success of the means of disinfection which we employ. The only conclusion that we ought at present to draw is, that it is of very great importance to extend our knowledge of azote and hydrogen. He adds, "I shall conclude with two observations which arise from these experiments, and which may show the utility of employing *colouring matters* as re-agents in chemical researches. We have only vague conjectures on the nature of different cutaneous secretions which take place in a great number of diseases. The chemical knowledge of these discharges will be of great service in medicine. The colouring matters offer themselves as useful instruments in this kind of discoveries. We only want an accurate account of the different changes of colour caused by the exhalations in a great many diseases, to institute a set of experiments, the results of which would be highly interesting."

---

Mon. Descroizilles has inserted some observations, in the Annales de Chimie, vol. lxvii. 80, on the use of the *pickle of violets*, as a re-agent, which can be had at all times, while the syrup is liable to be spoiled by keeping. It is made by pouring double their weight of boiling water upon the petals, slightly pressed, in a clean pewter measure, which is to be exposed for a few hours to a heat rather greater than a water bath. Add to the infusion, when strained, one third its weight of very fine white salt, stirring it till it is dissolved. It should be kept in a phial corked up. For use, dip a small stick into this liquid, and touch a clean earthen plate in several places. Vegetables and flowers, as roses, may be preserved any length of time for distillation by salting them. Rub one-third their weight of salt with them, which forms a kind of paste of little bulk; dilute this in the still with twice its weight of water, and the distilled liquor will be equally fragrant as when drawn from the fresh plants.

We have received a specimen of the gum of opium from Messrs. Vincent, Nurbel & Co. Chemists in Old Bond Street, which is much used in France, as possessing the anodyne and soporific qualities, without producing the disagreeable effects on the head and stomach, so much complained of in the common opium.— From the few trials we have made of this gum, it appears to be a valuable preparation of that important drug. One instance was that of a delicate lady, who had never been able to use common opium, even in small doses, without experiencing distressing nervous sensations, disturbed sleep, and on the morrow, severe headache and sickness. A grain of this gum, taken by her, produced none of these disagreeable effects. The dose is the same as that of common opium. The following is their method of separating the gum from the resin. Take any quantity of crude opium, and cut it it into small slices; pour a gallon of water to each pound of opium, and let it digest for forty-eight hours, often stirring the fluid. Filtre the liquor, and evaporate it to one half; to this add cold water, equivalent to what was evaporated. Filtre again on the morrow, and repeat this operation for ten days or more, or until the addition of cold water throws down no more precipitate; then evaporate the liquor in a water bath down to an extract. The resin precipitated by the cold water is kept for external applications.

The Austrian government has lately proposed the following prize-questions, relative to substitutes for various foreign articles in the Materia Medica. 1. What indigenous or European productions, distinguished by specific effects, may be substituted for those now brought from India. 2. A substitute for camphor. 3. A substitute for Peruvian bark. 4. What species of plants may replace senna, jalap, and ipecacuanha? 5. A substitute for opium. The prize for each question is five hundred ducats.

The Medical Society of London, held their Anniversary Meeting on the 8th instant, at their house in Bolt-court, when the following officers were elected. President, Dr. Lettsom; Vice-Presidents, Dr. Bancroft, Mr. Norris, Dr. Pinckard and Mr. Abernethy; Librarian, Dr. Clutterbuck; Secretaries, Mr. Good and Dr. Hamilton; Council, Mr. Field, Mr. Chamberlaine, Dr. Haighton, Dr. Adams, Mr. Seaton, Dr. Thornton, Mr. Griffith, Mr. Robinson, Mr. A. Cooper, Mr. M'Donald, Mr. Hopkins, Mr. Geo. Young, Mr. Platt, Mr. Addington, Dr. Lidderdale, Mr. Ramsden, Dr. N. E. Bancroft, Mr. Lawrence, Dr. Shearman, Mr. Forster, Mr. Saumarez, Mr. Lewis, Mr. Upton, Mr. Beveridge, Mr. Harvey, Dr. Temple, Dr. Bateman, Dr. Fothergill, Dr. Birkbeck, and Dr. Hancock; Registrar, Mr. A. B. Turnbull. After which the Society adjourned to the London Coffee-house, Ludgate-hill where an excellent oration was delivered to a numerous audience by Dr. Birkbeck, "on the inaccuracy of Medical Logic, and on the Expectation of future Improvements in the Science of Medicine." The Members of the Society afterwards dined together as usual.

Dr. Bradley is preparing a Work, to contain the First Lines of the London Practice of Physic. This is intended to be a strictly practical book, and therefore, will not include those preparatory branches, denominated the Institutions by Boerhave, or the Theory of Medicine by others. It is also designed to avoid all interference with Midwifery and Surgery; so that one moderate volume, it is presumed, may contain the first lines of medical practice. The Author will avail himself of the assistance, both of his medical friends in the metropolis, and of the labours of those who have practised in distant countries; or where climate has a powerful effect in modifying the symptoms and mode of treatment.

---

We are much obliged to Mr. Foster for his communication, which, with Mr. Hargrove's, shall appear in our next.

